# Correlations between Antioxidant and Biochemical Parameters of Blood Serum of Duroc Breed Pigs

**DOI:** 10.3390/ani11082400

**Published:** 2021-08-13

**Authors:** Sergei Yu. Zaitsev, Anna A. Belous, Oksana A. Voronina, Roman A. Rykov, Nadezhda V. Bogolyubova

**Affiliations:** Federal Research Center for Animal Husbandry Named after Academy Member L.K. Ernst, Dubrovitsy 60, 142132 Moscow, Russia; belousa663@gmail.com (A.A.B.); voroninaok-senia@inbox.ru (O.A.V.); brukw@bk.ru (R.A.R.); 652202@mail.ru (N.V.B.)

**Keywords:** Duroc breed boars, antioxidants, biochemistry, blood parameters, feeding time, correlation coefficients

## Abstract

**Simple Summary:**

Human nutrition is currently one of the most important factors that determine health, performance, duration and quality of life. The increasing demand for high-quality livestock products requires scientists and practitioners to develop an advanced complex approach to assessing the composition of animal meat and methods of its regulation. One of these products is pork, the value of which lies primarily in the lipid and protein content, which are necessary for human nutrition. The significance of this paper is also determined by the high popularity of pork in Russia and in a number of other countries worldwide.

**Abstract:**

Correlations between the major biochemical (BC) and antioxidant (TAWSA) parameters of pigs’ blood are necessary to study in order to assess physiological–biochemical status (PhBS), animal health, production, etc. Blood samples were obtained from Duroc breed boars (*n* = 77), divided into groups 1 (*n* = 25), 2 (*n* = 40) and 3 (*n* = 12), which were fattened for 65, 72 and 100 days, respectively. Significant positive and negative correlations were found between TAWSA and BC parameters of pigs’ blood for group 3: very high in the case of total protein (TP) (−0.75) and aspartate aminotransferase (AST) (−0.79); high in the case of cholesterol (−0.72), glucose (0.66), alkaline phosphatase (0.66), calcium ions (−0.60) and globulins (0.53); moderate in the case of albumins (−0.36), triglycerides (−0.35), magnesium (−0.32) and phosphorus (−0.27). The same was found for group 2: high in the case of TP (0.51); moderate in the case of globulins (0.48), cholesterol (0.33) and phosphates (0.25). The only moderate correlation was found for group 1: magnesium (−0.48), glucose (0.36) and calcium (−0.25). This tendency indicated the stabilization of pig PhBS during growth and fattening, which can be useful for understanding the PhBS and antioxidant features of pigs, the factors of their nutrition, maintenance, etc.

## 1. Introduction

It is known that animal products are important sources of high-quality proteins, fats, vitamins, minerals and macro- and microelements in human nutrition [1]. In this regard, some approaches have been developed to improve the introduction of animal breeding [2,3,4] in order to ensure a higher production efficiency and meat quality (including pork), which is especially important for Russia [2,4,5,6]. Duroc is one of the most popular breeds of pig, possessing numerous positive characteristics in growth rate; total body weight; chemical composition of meat and fat, i.e., high intramuscular fat content in meat [6,7,8].

To assess the physiological–biochemical status (PhBS) and animal health, safety and quality of meat products [8,9,10], a complex of biochemical and hematological, antioxidant and “zootechnical” indicators must be studied.

To date, the PhBS of pigs, taking into account the changes in the content of antioxidants in the blood and other tissues of fattening animals, has not been sufficiently studied (both in the Russian Federation and in Asian countries) even for purebred animals. The assessment of PhBS of breeding pigs in Russia is mainly based on the study of changes in biochemical parameters in connection with feeding conditions [11,12,13], genotype [14,15], sex [16], age [17] and others (only some Russian works is cited as references in this part of the paper). For example, there was an interesting study on selective biochemical and hematological parameters of blood, as well as indicators of mineral metabolism in pigs of the Landrace and Kemerovo breeds, fed at large industrial enterprises of the “Chistogorsk” and “Altaymyasoprom” complexes in Russia [18]. These indicators are close to those obtained in our study and are within physiological norms [18].

On the other hand, some hematological and biochemical parameters of blood were studied in novel hybrids of boars (Large White Landrace) [19]. In industrial combinations (three breeds), where the meat breeds Pietrain and Duroc were used, the content of hemoglobin and erythrocytes was higher as compared to purebred animals [19]. From a practical point of view, data on the relationship of blood biochemical parameters with fattening and meat qualities [20,21,22] are especially important. There are a number of dissertations and articles on the assessment of PhBS in pigs of the Russian population based on the biochemical parameters of blood and meat of animals [4,9,17,23,24], which we will discuss in detail in the part 4 of our article. The least studied issue in this area is the study of the total amount of water-soluble antioxidants (TAWSA) in the blood serum of fattening pigs. In Russia in this area, only the groups from our Federal Research Center work in collaboration with physiologists and livestock specialists [24,25,26]. At the same time, individual indicators of antioxidant protection (for example, a concentration of TBA-active products) have been studied more fully [26,27] than TAWSA, but do not provide a complete knowledge of the antioxidant status of the pigs’ organisms.

Some authors [8,9,10,28] believe that the assessment of PhBS through the specified set of indicators is very important, and “the average population values of biochemical parameters are the starting point for the subsequent monitoring of breeding populations” [28]. For example, using these data, it is possible to analyze “in which direction the biochemical status of the population changes when pigs are selected for various characteristics of productivity” and to carry out the ecological monitoring of populations of certain breeds of pigs “in zones with different anthropogenic pressures” [8,9,10,28]. In addition, the obtained data may be the “average population norm” for healthy animals [28].

The studies carried out to assess the PhBS and health of animals of the Duroc breed, which showed a comparison of hematological and biochemical parameters (more than 40 parameters) between purebred and crossbred offspring [29]. The data obtained are consistent with previous studies [30] on crossbred and purebred offspring, in which there were small differences in some biochemical parameters of pigs at 15 and 27 weeks [30], including creatine, alkaline phosphatase, phosphorus and calcium [29], and were “corrected” with the further growth of animals [29,30].

For a correct assessment of PhBS, it is necessary to study the possibilities of protecting the body from reactive oxygen species (ROS) [31,32,33]. ROS are formed during many metabolic processes in human and animal cells, capable of oxidizing biologically active compounds (BAC), and damaging the membranes and cells of the body [31,32,33]. The body’s antioxidant defense system is designed to maintain the balance of bioactive substances (lipids, peptides, vitamins and other compounds) in organs and tissues of humans and animals, protecting them from ROS [34,35,36]. Antioxidant activity is a valuable source of information about the state of health and the level of stress resistance in humans and productive animals in industrial conditions [34,35,36], which led to a huge variety of methods for its study [33,36]. All these methods are based on a model reaction (most often oxidation) of an individual compound proceeded by a radical mechanism [33,34,35,36]. Among these, one of the relatively simple and reliable methods is the electrochemical method based on the amperometric detection of the oxidation reaction signal [33,34,35,36].

Duroc is one of the most popular breeds in Russia and Asian countries (including China), which is used not only for meat production, but also for reproduction in pig breeding. It is important to highlight that pork is one of the key directions in Russian livestock production.

In connection with the above-stated relevance, the aim of the work was to study the biochemical and antioxidant parameters of the blood of Duroc breed boars of the Russian population and to identify the most significant correlations between these parameters. These data will be used by the Ministry of Agriculture of the Russian Federation and are especially important in order to establish the range of particular biochemical references (standard norms) for the Duroc breed of pigs in the Russian population.

## 2. Materials and Methods

### 2.1. The Samples of the Pig Blood Serum

The studies were carried out on the basis of a selection-hybrid center on boars of the Duroc breed (*n* = 77), from which blood samples were taken from the ear vein during setting and withdrawing from fattening. These pigs were divided onto groups 1 (*n* = 25), 2 (*n* = 40) and 3 (*n* = 12) that were fattened for 65, 72 and 100 days, respectively. The age of boars’ setting at the feeding stations was 75 ± 1 days, with an initial live weight of approximately 35.0 ± 0.5 kg. The times of boars’ withdrawing were from 130 to 175 days, the final live weights were from 92 to 122 kg. Only clinically healthy animals that were periodically examined by veterinary specialists participated in the studies.

The experimental protocols concerning these animals were approved by the Bioethical Committee of the Federal Research Center for Animal Husbandry named after Academy Member L.K. Ernst. All experiments and conditions (animal care, feeding, biological material sampling, etc.) are fulfilled in accordance with the applicable regulations (internationally recognized guidelines and local acts).

### 2.2. Measurements of the Biochemical Parameters of Pig Blood Serum Samples

The biochemical parameters of animal blood serum samples were determined using a “ChemWell” automatic biochemical analyzer (Awareness Technology, Palm City, FL, USA). The open system of this analyzer allows the use of any method or reagent. Therefore, in this study, we used the reagents “Analyticon Biotechnologies AG” (Lichtenfels, Germany) and “Spinreact” (Carretera Santa Coloma, Spain). All reactions were performed in standard “microwell” plates. Pre-dilution, mixing, incubation, rinsing and measurement of the samples were controlled automatic. The following biochemical indicators were determined [37,38,39]: the concentration of total protein (TP)—by the biuret method; albumin (A)—by the colorimetric method with bromcresol green; urea—by enzymatic colorimetric analysis (Berthelot method); creatinine—by the kinetic Yaffe method; glucose—by the enzymatic glucose oxidase method; cholesterol (Chol) and triglycerides (TG) by the enzyme-colorimetric method; bilirubin (quantification by the Walters and Gerarde method); calcium (Ca)—by the O-cresolphthalein complexon method; phosphorus (P), magnesium (Mg) and iron (Fe)—by the colorimetric method; alanine aminotransferase (ALT) activity—by the UV-kinetic method; aspartate aminotransferase (AST) activity by—the UV-kinetic method; alkaline phosphatase (ALP) activity—by the kinetic method. The following ratios and indicators were determined by calculation: A/G, Ca/P, ALT/AST and the concentration of globulins (G) [37,38,39]. The results of these measurements were statistically processed using the MS Excel program.

### 2.3. Measurements of the Total Amount of Water-Soluble Antioxidants of Pig Blood Serum Samples

The amperometric method [33,34,35,36,39] was used to study the total amount of water-soluble antioxidants (TAWSA). The measurements were carried out on a “Tsvet-Yauza 01-AA” device [39]. The TAWSA values were determined by measuring the strength of the electric current arising during the oxidation of molecules on the surface of the working electrode at a potential of ~500 mV. TAWSA was measured in equivalent to gallic acid as in reference [35]. For this, the “working solutions” were prepared from a gallic acid solution (100 mg/dm^3^) for calibration with a mass concentration of 0.2, 0.5, 1.0 and 4.0 mg/dm^3^. An amount of 2.2 mmol/dm^3^ phosphoric acid solution was used as an “eluent” [35,39]. The results of measuring the total antioxidant activity of the samples were statistically processed using the MS Excel program.

When analyzing the studied indicators, the methods of variation statistics were used to calculate the mean and standard error (M ± m) and the coefficient of variation (Cv,%), as well as performing correlation analysis using the STATISTICA 10 program (StatSoft, Moscow, Russia). 

The obtained datasets of the antioxidant and biochemical parameters boars of the Duroc breed (*n* = 77) are available online on the website of the L.K. Ernst Federal Research Center for Animal Husbandry (https://www.vij.ru/institut/struktura-organizatsii/nauchnye-podrazdeleniya/52-gruppa-analiticheskoj-biohimii accessed on 1 June 2021).

## 3. Results

The blood of animals has a complicated biochemical composition: proteins (including albumins, globulins and enzymes), lipids (triglycerides, cholesterol, etc.), minerals (calcium, magnesium, phosphate ions, etc.), etc. From the point of view of biological chemistry, blood serum is a multiphase colloidal system in which the main phase is aqueous moderate, and one of the important integral characteristics is the total amount of water-soluble antioxidants (TAWSA). The authors determined the main biochemical and antioxidant parameters of the blood serum of the Duroc breed of pigs, divided onto groups 1 (*n* = 25), 2 (*n* = 40) and 3 (*n* = 12), which were fattened for 65, 72 and 100 days, respectively (Table 1, Table 2 and Table 3).

By measuring the biochemical and antioxidant (TAWSA) blood parameters of the Duroc breed pigs (*n* = 77), the most significant differences between these parameters in connection with the days of animal fattening were revealed. It was shown that all biochemical and antioxidant parameters of the blood of pigs of both groups were within the physiological norms for this animal species. The main indicators of protein metabolism in the blood were fairly constant for groups 1 and 2, but changed significantly for group 3. Thus, the parameters of total protein (TP) and albumins (A) changed by less than 2% for groups 1 and 2, but significantly decreased (−7.1% and −11.9% for TP and A, respectively) for group 3 as compared to group 1 (Table 1). The content of globulins (G) varied in the range of +3.3% for group 2 up to −1.2% for group 3 versus group 1, respectively (Table 1).

It seemed logical that the A/G ratio changed by less than 1% for group 2 and more than 11.8% for group 3 (as compared to group 1); however, there was a significant decrease in the albumin values (−11.9%) for group 3 as compared with group 1, respectively, and insignificant changes in the globulin content for all the groups studied (Table 1, Table 2 and Table 3).

Some of these biochemical parameters differed slightly in a number of values: urea—by −5.3% and +4.7%, and creatinine—by −14.0% and −7.8%, for groups 2 and 3 compared to group 1, respectively (Table 1, Table 2 and Table 3). This is normal for a young growing organism and indicates an improvement in protein metabolism in a number of animals [37,38,39]. Similar values of the total protein content in the blood serum of piglets were also noted by other authors [9,10,37,38,39], and it was indicated that this parameter “differed by variability” [28].

On the other hand, a significant (more than −27.2% and −25.7%) decrease in pigs’ blood glucose values in groups 2 and 3 as compared to group 1 is surprising (Table 1, Table 2 and Table 3). Generally, with the increase in the age of pigs, there is a slight increase in the values of glucose in their blood. Therefore, in [28], it was found that the glucose content in piglets (at the age of 20–60 days) was at the level of 5.2–5.3 mmol/L, although the author pointed out the highest coefficient of variation (Cv = 42.2%) among all other biochemical parameters. In our studies, the coefficient of variation in the case of glucose ranged from 4% to 10%, which was typical for most other biochemical parameters.

It is interesting that for groups 1 and 2 (fed for 65 days and 72 days), such an important biochemical parameter of lipid metabolism as the content of triglycerides in the blood practically did not change, while the content of TG for group 3 animals (fed for 100 days) increased by 232%, i.e., almost 2.3 times (Table 1, Table 2 and Table 3).

The cholesterol content increased by 18.0% and 4.2% for groups 1 and 2 (Table 1, Table 2 and Table 3), respectively, which is indirect evidence of changes in lipid metabolism in a number of animals [39,40,41]. Some authors [9,10,39,40,41] note that at a young age, piglets of the Large White breed have the highest serum cholesterol content (for example, 4.21 ± 0.90 mmol/L at the age of 2–3 weeks [28], whereas by the age of two months, its content in piglets decreases to 2.70 ± 0.58 mmol/L and remains practically at the same level as in adult sows (2.67 ± 0.75 mmol/L) [28]. Of course, the coefficient of variation of this feature, according to the same authors [28], is a fairly large value of the order of 21–28%. These authors explain the tendency to a high cholesterol content in piglets by “more active metabolic processes in their body, including glycolysis” [28].

The following changes in enzyme activity were observed: ALT—by 21.0% and 17.1% (for groups 2 and 3) and AST—by −19% and approximately 0% (for groups 2 and 3), which did not go beyond the physiological norms. Since for such enzymes, only changes in values from 30% and higher are significant, only the change in the “de Ritis coefficient” should be considered, which decreased by more than −32% for the 2nd group of pigs as compared to the 1st and changed in the ALP activity by −37.0% for the 3rd group of pigs as compared to the 1st, respectively. Changes in ALP activity by −8.4% for the 2nd group of pigs as compared to the 1st are not significant (Table 1, Table 2 and Table 3).

The calcium content in the blood serum fell by −9.5% and −13.5%, whereas magnesium content sometimes increased by 10.2%, then fell by −24.2%, and the phosphorus content always increased by 17.6% and by 23.0% for the 2nd and 3rd groups of pigs as compared to the 1st one, respectively (Table 1, Table 2 and Table 3). These changes had a positive effect on the ratio of calcium to phosphorus in the blood serum of Duroc pigs.

Finally, the total amount of water-soluble antioxidants decreased by more than −23% in the 2nd group of pigs, and then increased by 96.1% in the 3rd group compared to the 1st group, respectively (Table 1, Table 2 and Table 3). This is directly related to the changes in a number of basic biochemical parameters of the blood of pigs of the Duroc breed, for example, albumin and TG listed above (Table 1, Table 2 and Table 3).

The experimental conclusions are summarized later. It should be noted that an increase in the duration of feeding (from 65 to 72 and 100 days) led to a tendency for a significant decrease in the coefficients of variation (Cv) for most biochemical parameters. This indicates the stabilization of the physiological and biochemical status of the growing organism in the 2nd and 3rd pig groups in comparison with the 1st one (Table 1, Table 2 and Table 3).

## 4. Discussion

### 4.1. The Relationship between the Biochemical Parameters of the Blood Serum of Boars of the Duroc Breed

In recent years, lipid peroxidation has become the subject of extensive research in terms of mechanisms, dynamics, product analysis, disease involvement, inhibition and biological signaling. Some types of antioxidants with different functions inhibit lipid peroxidation and the harmful effects caused by lipid peroxidation products. Much attention has recently been paid to the biological role of lipid peroxidation products, but it is topical to study the relationship between biochemical parameters and indicators of antioxidant protection [38,42].

The relationships of biochemical parameters in group 1 are presented in Table S1 (65 days of fattening, *n* = 25). The presence of 5 very strong, 5 strong (i.e., subtotal—10 significant) and 36 moderate correlations (i.e., subtotal—46 meaningful) from the 136 total independent correlations was found. In particular, there were only 2 very strong, 1 strong and 16 moderate correlations between the 4 protein indicators in group 1 (i.e., correlations—8 for TP, 6 for albumins and 5 for globulins) correlations from the 58 total independent correlations. There was only 1 very strong correlation between enzymes and 2 strong correlations between enzymes and inorganic ions, as well as 2 strong correlations between cholesterol and inorganic ions. There were only 2 very strong correlations between inorganic ions, as well as numerous moderate correlations in the case of glucose, triglycerides, cholesterol, enzymes, magnesium ions, phosphates, inorganic ions and the Ca/P ratio.

The relationships of biochemical parameters in group 2 are presented in Table S2 (72 days of fattening, *n* = 40). The presence of 4 very strong, 11 strong (i.e., subtotal 15 significant) and 37 moderate (i.e., subtotal 52 meaningful) correlations from the 136 total independent correlations was found. In particular, there were only 3 very strong, 7 strong and 16 moderate correlations between the 4 protein indicators in group 2 (i.e., correlations—9 for TP, 7 for albumins, 5 for globulins and 3 for the A/G ratio) from the 58 total independent correlations. There was only 1 very strong correlation between enzymes and 3 strong correlations between inorganic ions, as well as 1 strong correlation between urea and Mg^2+^ ions. There were numerous moderate correlations in the case of glucose, triglycerides, cholesterol, enzymes and magnesium ions with Ca/P ratio.

The relationships of biochemical parameters in group 3 are presented in Table S3 (100 days of fattening, *n* = 12). The presence of 12 very strong, 28 strong (i.e., subtotal 40 significant) and 47 moderate correlations (i.e., subtotal 87 meaningful) from the 136 total independent correlations was found. In particular, there were 6 very strong, 9 strong and 20 moderate correlations between the 4 protein indicators in group 3 (i.e., correlations—11 for TP, 9 for albumins, 10 for globulins and 5 for the A/G ratio) correlations from the 58 total independent correlations. There were 6 very strong, 19 strong and numerous moderate correlations in the case of all other organic compounds and inorganic ions, as well as their ratios.

Thus, animal group 3 was preferential for blood biochemistry correlations as compared to groups 1 or 2. There were 2.5–3.0 times more very strong correlations, 2.5–5.6 times more strong correlations and approximately 1.3 times more moderate correlations in the case of animal group 3 (in total) as compared to the groups 1 or 2. Moreover, there were 2.0–3.0 times more very strong correlations, 1.3–9.0 times more strong correlations and approximately 1.25 times more moderate correlations between the 4 protein indicators in the case of animal group 3 (in total) as compared to the groups 1 or 2. It is important to highlight that there were no meaningful correlations of the total protein indicator and its fractions with the A/G ratio for group 3 as compared to groups 1 or 2.

It is well known [42,43,44,45,46] that the PhBS of animals is initially determined by the multilevel and complex interaction of the systems of the animal body. Therefore, a comparative analysis of the biochemical parameters of blood is especially important. For example, according to Molyanova G.V. [17], the total protein content in pigs’ blood at the age of 120 days is 61.12 or 62.02 g/L and at the age of 180 days—63.02 or 72.05 g/L (for Duroc breed pigs in Samar region) in the cold or warm weather periods, respectively. According to Giro T.M. et al. [44], the total protein content in pigs’ blood at the age of 120 days is approximately 74.6 g/L; albumins—38.9 g/L; globulins—35.5 g/L (for Duroc breed pigs in Saratov region). In the work of Nikolaev D.V. et al. [45], the level of total protein in pigs’ blood at the age of 180 days is 78.5 g/L; albumins—33.7 g/L; globulins—44.8 g/L (for Duroc breed pigs in the Volgograd region). We compared the data of 11 biochemistry indicators (total protein, albumin, globulins, A/G, glucose, triglycerides, ALT, AST, ALP, Ca and P) from all of these studies, which have different values between them, with our results (for Duroc breed pigs in the Voronezh region), but within the range of general data of blood biochemistry for healthy pigs [38]. This is why a detailed study on the major biochemical parameters of the blood serum of such pigs is especially important in order to establish the range of particular biochemical norms for the Duroc breed pigs in the Russian population.

The data of biochemical analysis, obtained in the work of Gu T. [46], did not show significant differences in more than 40 parameters in purebred Duroc pigs, their clones and offspring (including two-breed hybrids in various variations). According to Gu T. et al. [46], on the 112th day, the level of total protein in the blood of Duroc pigs was 76.38 g/L; albumin—40.08 g/L; globulins—36.30 g/L; glucose—3.35 mmol/L; calcium—2.65 mmol/L. However, in the work of Gu T. [46], we did not find data characterizing the state of the antioxidant system in pigs. At the same time, the activity of enzymes such as ALT, AST and ALP is more variable and can vary significantly. Thus, in our study, ALT and AST activity was observed up to 30 U/L for all studied groups of animals (from 140 days of fattening to 175 days), while in [19], the ALT activity was 91.66 U/L and AST activity was 89.63 U/L, which is within the normal range (the norms for ALT 22–98 IU/L and AST 13–95 IU/L according to Gusev I.V. [39]). The data of biochemical analysis, which we obtained in the study of animals of the Duroc breed, are in good agreement with the data obtained in the above works [44,45,46], and there are also relatively close values in the levels of total protein, glucose and calcium content in [19] for three-breed hybrids. As a literature search showed, most of the works are focused on biochemical parameters, while scarce attention is paid to the study of antioxidant systems in a comprehensive assessment of health status. Of course, the rate of metabolic processes in the body directly affects the formation of free radicals and their neutralization by both enzymatic and low molecular weight antioxidants. The assessment of these processes is extremely important, as confirmed by the work of Kotenkova E.A. [47], which studied the antioxidant potential of the pig spleen, heart and aorta extracts (by determining their total antioxidant capacity after slaughter). The highest total antioxidant capacity was observed in the spleen extract. However, no attention was paid to blood in the context of this work, which could be interesting for the development of a strategy to increase the antioxidant activity of food products.

### 4.2. The Relationship of TAWSA with Biochemical Parameters of the Blood Serum of Boars of the Duro Breed

For the first time, the calculation of phenotypic correlations of biochemical and antioxidant parameters of the blood of pigs of the Duroc breed was carried out (Table 4).

As shown in our previous works on the study of correlations between TAWSA and biochemical parameters of sheep blood [38], positive or negative values of correlations are not as important as their absolute values, i.e., whether these correlations are very strong (0.75–1.0), strong (0.50–0.74), moderate (0.25–0.49) or weak (0.01–0.24) [38]. In the latter case (weak correlations), it makes no sense to discuss their direction, i.e., whether they are positive or negative [38]. Here, we focused on describing very strong, strong and moderate correlations (in this sequence mainly) between the studied parameters.

Significant positive and negative correlations were found between TAWSA and the following biochemical parameters of pigs’ blood for group 3: very high in the case of TP (−0.75) and AST (−0.79); high in the case of cholesterol (−0.72), glucose (0.66), alkaline phosphatase (0.66), calcium ions (−0.60) and globulins (0.53); and moderate in the case of albumin (−0.36), triglycerides (−0.35), magnesium ions (−0.32) and phosphorus (−0.27) (Table 4).

Significant positive correlations were found between the following biochemical and antioxidant parameters of pigs’ blood for group 2 (Table 4): high in the case of TP (0.51) and moderate in the case of globulins (0.48), cholesterol (0.33) and phosphates (0.25).

The correlation between TAWSA and biochemical parameters was the most significant only in the case of magnesium ions (−0.48), glucose (0.36) and calcium ions (−0.25) for group 1 (Table 4), i.e., only moderate correlations were found.

Significantly higher values of the correlation coefficients between TAWSA and biochemical parameters (Table 4) and a large number of significant correlations were obtained for group 3 (11 in total, including 7 strong and very strong) as compared to groups 2 (4 in total, including 1 strong) and 1 (3 in total, only moderate). Thus, a clear tendency indicated the stabilization of the PhBS of animals during their growth and fattening. The same dependences concerning correlations of TAWSA and the biochemical parameters of pigs’ blood were found in our ongoing research of hybrid animals (Duroc, Landras and Large White pigs of the Russian population).

It is important to highlight that the pronounced correlations between antioxidant and biochemical blood parameters were found earlier in our previous research in hybrid sheep breeds [38] and only in a short presentation concerning different sheep breeds [43]. For example, the author of [43] found the relationship of lipid peroxidation (LPO) parameters with some hematological and biochemical blood parameters of the following breeds: Texel x Manych Merino, Grozny Small-haired Merino and Soviet Merino. At a high level of lipid peroxidation (5.2 mmol/L), an inverse correlation was established among hemoglobin, total protein and the gamma-globulin fraction of protein for crossbred animals [43]. The high (r = −0.6–0.7) and very high (r = −0.9) negative correlations were revealed between LPO and total proteins (or albumins) for Soviet merino at the same antioxidant background (MDA = 5.0 mmol/L) [43]. Significant correlations were also established for the Grozny Yark breed (r = 0.8–0.7 for hemoglobin and albumin and r = −0.9 for globulins) [43]. All of these data indicated a particular balance between oxidative and reduction processes in the body of these sheep breeds, as considered by the authors of [34,35,38,43].

## 5. Conclusions

The presented data on the biochemical and antioxidant parameters of the blood serum of the Duroc breed pigs and their specific correlations were obtained for the first time. The tendency towards the optimization of blood biochemical parameters in adult animals can be useful for understanding the features of PhBS and the antioxidant status of pigs. The revealed tendencies can be explained only by a closer (than previously thought) relationship between the biochemical and antioxidant functionality of the pig body. In our opinion, the rate of ROS neutralization can be related to the rate of reaching a live weight of 100 kg, which is an important economic feature. This can be explained by the fact that the metabolic energy with an increase in the fattening period will be directed mainly to gaining muscle mass, but not “to fight free radicals” (ROS). The authors assume that the revealed tendency of changes in the biochemical and antioxidant parameters of Duroc breed pigs (at a longer feeding duration) will be monitored in our further ongoing experiments with hybrid animals. These data will be used by the Ministry of Agriculture of the Russian Federation and are especially important in order to establish the range of particular biochemical references (standard norms) for the Duroc breed pigs of the Russian population.

## Figures and Tables

**Table 1:** Biochemical and antioxidant parameters of the blood serum of the Duroc breed pigs (*n* = 25) in group 1 (65 days of fattening).

Parameters ^1^	Mean Value ^1^	Standard Deviation ^2^	Error ^1^	Coefficient of Variation ^3^
Total protein, g/L	75.29	4.44	0.91	5.89
Albumin, g/L	41.60	3.56	0.73	8.56
Globulin, g/L	33.70	5.24	1.07	15.55
A/G	1.27	0.24	0.05	19.06
Urea, mM/L	7.60	1.63	0.33	21.41
Creatinine, μM/L	112.88	21.46	4.38	19.00
Glucose, mM/L	5.33	0.55	0.11	10.27
Triglycerides, mM/L	0.28	0.02	0.01	8.06
Cholesterol, mM/L	2.12	0.25	0.05	11.67
ALT, IU/L	25.03	8.48	1.73	33.88
AST, IU/L	29.60	13.85	2.82	46.79
De Ritis coefficient AST/ALT	1.20	0.54	0.11	45.00
Alk. phosphatase, IU/L	187.91	45.77	9.3	24.36
Ca, mM/L	2.74	0.22	0.05	8.08
P, mM/L	2.96	0.38	0.08	12.88
Mg, mM/L	1.28	0.17	0.04	13.42
TAWSA, mg/L	9.77	3.01	0.61	30.81

^1^ same unit as mentioned in the “Parameters” (i.e., g/L, mM/L, μM/L, etc.); ^2^ rel. units; ^3^ %.

**Table 2:** Biochemical and antioxidant parameters of the blood serum of the Duroc breed pigs (*n* = 40) in group 2 (72 days of fattening).

Parameters ^1^	Mean Value ^1^	Standard Deviation ^2^	Error ^1^	Coefficient of Variation ^3^
Total protein, g/L	76.76	8.30	1.31	10.81
Albumin, g/L	41.93	4.59	0.73	10.90
Globulin, g/L	34.80	6.84	1.08	19.66
A/G	1.26	0.30	0.05	23.81
Urea, mM/L	7.20	0.99	0.16	13.75
Creatinine, μM/L	97.08	20.91	3.31	21.54
Glucose, mM/L	3.88	0.99	0.16	25.52
Triglycerides, mM/L	0.28	0.02	0.01	7.14
Cholesterol, mM/L	2.50	0.47	0.07	18.80
ALT, IU/L	30.39	6.56	1.04	21.59
AST, IU/L	24.04	11.49	1.82	47.80
De Ritis coefficient AST/ALT	0.81	0.34	0.05	41.98
Alk. phosphatase, IU/L	172.17	52.00	8.22	30.20
Ca, mM/L	2.48	0.23	0.04	9.27
P, mM/L	3.48	0.36	0.06	10.34
Mg, mM/L	1.15	0.14	0.02	12.14
TAWSA, mg/L	7.50	1.77	0.28	23.60

^1^ same unit as mentioned in the “Parameters” (i.e., g/L, mM/L, μM/L, etc.); ^2^ rel. units; ^3^ %.

**Table 3:** Biochemical and antioxidant parameters of the blood serum of the Duroc breed pigs (*n* = 12) in group 3 (100 days of fattening).

Parameters ^1^	Mean Value ^1^	Standard Deviation ^2^	Error ^1^	Coefficient of Variation ^3^
Total protein, g/L	69.95	4.03	1.52	5.76
Albumin, g/L	36.66	2.13	0.99	5.81
Globulin, g/L	33.20	4.25	1.11	12.76
A/G	1.12	0.21	0.09	18.75
Urea, mM/L	7.96	1.21	0.26	15.20
Creatinine, μM/L	104.14	15.64	0.93	15.02
Glucose, mM/L	3.96	0.76	0.14	19.19
Triglycerides, mM/L	0.93	0.09	0.10	9.68
Cholesterol, mM/L	2.21	0.21	0.96	9.50
ALT, IU/L	29.31	5.64	0.86	19.24
AST, IU/L	29.61	8.73	0.96	29.48
De Ritis coefficient AST/ALT	1.05	0.35	0.49	33.33
Alk. phosphatase, IU/L	118.36	39.54	1.58	33.41
Ca, mM/L	2.37	0.35	0.92	14.77
P, mM/L	3.64	0.27	0.09	7.42
Mg, mM/L	0.97	0.06	0.08	6.19
TAWSA, mg/L	19.16	2.44	1.07	12.73

^1^ same unit as mentioned in the “Parameters” (i.e., g/L, mM/L, μM/L, etc.); ^2^ rel. units; ^3^ %.

**Table 4:** Correlations of antioxidant parameters (TAWSA) with biochemical parameters of the blood serum of the Duroc breed pigs in groups 1, 2 and 3 (65, 72 and 100 days of fattening, respectively).

Parameters	Group 1, *n* = 25 (65 Days of Fattening).	Group 2, *n* = 40 (72 Days of Fattening).	Group 3, *n* = 12 (100 Days of Fattening).
Total protein, g/L	−0.15	0.51	−0.75
Albumin, g/L	0.00	0.21	−0.36
Globulin, g/L	−0.12	0.48	−0.53
Urea, mM/L	0.23	0.20	0.26
Creatinine, μM/L	0.23	0.20	−0.18
Glucose, mM/L	0.36	−0.09	0.66
Triglycerides, mM/L	0.24	0.09	−0.35
Cholesterol, mM/L	0.21	0.33	−0.72
ALT, IU/L	0.20	0.21	0.13
AST, IU/L	−0.16	0.16	−0.79
Alk. phosphatase, IU/L	−0.03	−0.19	0.66
Ca, mM/L	−0.25	−0.05	−0.60
P, mM/L	−0.12	0.25	−0.27
Mg, mM/L	−0.48	0.37	−0.32

## Data Availability

Data supporting reported results can be found at: https://www.vij.ru/goszadanie-i-proekty/proekty/proekty-rnf/749-proekt-20-16-00032-rnf (accessed on 1 June 2021).

## Supplementary Materials

The following are available online at www.mdpi.com/article/10.3390/ani11082400/s1, Table S1: Correlations of biochemical parameters* of the blood serum of the Duroc breed pigs in group 1 (65 days of fattening), Table S2. Correlations of biochemical parameters of the blood serum of the Duroc breed pigs in group 2 (72 days of fattening), Table S3. Correlations of biochemical parameters of the blood serum of the Duroc breed pigs in group 3 (100 days of fattening).

## References

[1] Ernst L.K., Zinovieva N.A. (2008). Biological Problems of Animal Husbandry in the XXI Century.

[2] Belous A.A., Trebunskikh E.A., Kostyunina O.V., Sermyagin A.A., Zinovieva N.A. (2019). Evaluation of signs of feed conversion and feeding behavior of Duroc boars using automatic feeding stations. Achiev. Sci. Technol. Agro-Ind. Complex.

[3] Colpoys J.D., Johnson A.K., Gabler N.K. (2016). Daily feeding regimen impacts pig growth and behavior. Physiol. Behav..

[4] Neupokoeva A.S. (2019). Productive and Biological Qualities of Pigs of Canadian Selection in the Conditions of the Trans-Urals. Ph.D. Thesis.

[5] Aaslyng M.D., Oksama M., Olsen E.V., Bejerholm C., Baltzer M., Andersen G., Bredie W.L.P., Byrne D.V., Gabrielsen G. (2007). The impact of sensory quality of pork on consumer preference. Meat Sci..

[6] Nevrkla P., Kapelański W., Václavková W.E., Hadaš Z., Cebulska A. (2017). Meat quality and fatty acid profile of pork and backfat from an indigenous breed and a commercial hybrid of pigs. Ann. Anim. Sci..

[7] Velichko V.A., Patieva A.M. (2011). The influence of the genotype on the nutritional value of pig meat. Proc. Kuban State Agrar. Univ..

[8] Burmistrov V., Pustovit I. (2005). Physico-chemical composition of muscle and adipose tissue in pigs of different genotypes. Pig Breed..

[9] Dementyeva T.A. (1998). Characteristics of the Productivity of Pigs by Biochemical and Cytochemical Tests in Purebred Breeding and Crossbreeding. Ph.D. Thesis.

[10] Pond W.G., Houpt K.A. (1983). The Biology of the Pig, Transl. from English, V.V. Popova.

[11] Shalak M.V., Portnoy A.I., Katushenok N.N. (2010). Aromatic additive in the diet of fattening pigs and its effect on morphological and biochemical blood parameters. Sci. Notes Educ. Inst. Vitebsk Order Badge Honor State Acad. Vet. Med..

[12] Yunusova O.Y. (2017). The influence of the feed additive “kostovit-forte” on the morphological and biochemical parameters of the blood of pigs on fattening. Perm Agrar. Bull..

[13] Serdyukova Y.A., Zlepkin V.A. (2016). The effect of feed additives on morphological and biochemical blood parameters of fattened pigs. Bull. Agro-Ind. Complex Stavropol.

[14] Kosilov V.I., Perevoiko Z.A. (2015). Biochemical parameters of blood serum of young pigs of large white breed of different genotypes. Proc. Orenbg. State Agrar. Univ..

[15] Garskaya N.A., Peretyatko L.G. (2019). Some biochemical blood parameters and their variability in boars of the Poltava meat breed depending on the genotype. Genet. Anim. Breed..

[16] Perevoiko Z.A., Kosilov V.I. (2014). Basic biochemical blood parameters of boars and sows of large white breed. Proc. Orenbg. State Agrar. Univ..

[17] Molyanova G.V. (2011). Formation of the Physiological Immune Status Pigs with Age Depending on Heliogeophysical and Climatic Factors the Middle Volga Region and It’s Correction with Thymosin-α1. Ph.D. Thesis.

[18] Sebezhko O.I., Korotkevich O.S., Konovalova T.V., Biryulya I.K., Petukhov V.L., Kamaldinov E.V., Osadchuk L.V. Biochemical, hematological and mineral parameters in pigs of two breeds reared in large industrial complexes of Western Siberia. Proceedings of the 3rd International Symposium for Agriculture and Food–ISAF 2017.

[19] Chernev I.F. (2021). Influence of boor genotype on hematological and biochemical indicators of blood in hybrid pigs. Anim. Breed. Genet..

[20] Shakhbazova O.P. (2011). Biochemical blood parameters and their relationship with fattening and meat qualities in pigs of different genotypes. Vet. Pathol..

[21] Maksimov G.V., Polozyuk O.N. (2011). The relationship of meat productivity with biochemical parameters of blood of pigs of different genotypes according to the RYR-1 gene. Bull. Don State Agrar. Univ..

[22] Halak V.I., Chernyavsky S.E., Ilchenko M.A., Petulko P.V., Gorchanok A.V., Shebeko K.K., Volkova E.M., Kruchinsky N.G., Nikandrov V.N., Cheshchevik V.T. (2019). Biochemical parameters of blood serum and their relationship with fattening and meat qualities of young pigs of different genotypes according to snp c. 1426 g>a of the melanocortin 4 receptor gene (MC4R). Biotechnology: Achievements and Development Prospects. Collection of Materials of the IV International Scientific and Practical Conference.

[23] Kireev I.V., Orobets V.A., Sevostyanova O.I., Shakhova V.N., Agarkov A.V. (2018). Prospects of using antioxidant drugs for the treatment and prevention diseases of farm animals. Res. J. Pharm. Biol. Chem. Sci..

[24] Magomedaliev I.M., Nekrasov R.V., Chabaev M.G., Dzhavakhiya V.V., Glagoleva E.V., Kartashov M.I., Durnikin D.A., Matsyura A.V. (2019). Use of different concentrations of Enzymesporin probiotic in feeding of growing young pigs. Ukr. J. Ecol..

[25] Fomichev Y.P., Bogolyubova N.V., Nekrasov R.V., Chabaev M.G., Rykov R.A., Semenova A.A. (2020). Physiological and biochemical effects of two feed antioxidants in modeling technological stress in pigs. (Sus scrofa domesticus Erxleben, 1777). Biol. Agric..

[26] Nikanova L.A., Fomichev Y.P. (2016). Bioprotective effect of dihydroquercetin and arabinogalactan in weakening the influence of extreme environmental factors on the body of pigs. Collect. Sci. Pap. North Cauc. Res. Inst. Anim. Husb..

[27] Lyubin N.A., Stetsenko I.I., Lyubina E.N. (2013). Functional state of the system of antioxidant protection and free radical oxidation in pigs depending on the use of various forms of vitamin A and beta-carotene. Bull. Ulyanovsk State Agric. Acad..

[28] Tyan E.A. (2004). Biochemical status of pigs of large white breed of Western Siberia. Successes Mod. Nat. Sci..

[29] Maja W.I., Øyvind N., Gjerlaug-Enger E., Eli G., Marcos S.L., Theo M. (2019). Effects of heterozygosity on performance of purebred and crossbred pigs. Genet. Sel. Evol..

[30] Maselyne J., Saeys W., Van Nuffel A. (2015). Review: Quantifying animal feeding behavior with a focus on pigs. Physiol. Behav..

[31] Halliwell B., John M.C. (2015). Free Radicals in Biology and Medicine.

[32] Bohn T. (2019). Carotenoids and Markers of Oxidative Stress in Human Observational Studies and Intervention Trials: Implications for Chronic Diseases. Antioxidants.

[33] Yashin Y.I., Ryzhnev V.Y., Yashin A.Y., Chernousov N.I. (2009). Natural Antioxidants. Content in food Products and Their Impact on Human Health and Aging.

[34] Savina A.A., Voronina O.A., Bogolyubov N.V., Zaitsev S.Y. (2020). Amperometric detection of antioxidant activity of model and biological fluids. Bull. Mosc. Univ. Chem..

[35] Voronina O.A., Savina A.A., Bogolyubov N.V., Zaitsev S.Y. (2019). The total amount of water-soluble antioxidants in the blood serum of productive animals. Veterinary Medicine. Anim. Sci. Biotechnol..

[36] Yashin A.Y. (2008). Injector-Flow System with Amperometric Detector for Selective Determination of Antioxidants in Meal Additives and Drinks. Russ. Chem. J..

[37] Zaitsev S.Y., Bogolyubov N.V., Zhang X., Brenig B. (2020). Biochemical parameters, dynamic tensiometry and circulating nucleic acids for cattle blood analysis. PeerJ.

[38] Zaitsev S.Y., Savina A.A., Volnin A.A., Voronina O.A., Bogolyubova N.V. (2020). Comparative Study of the Water-Soluble Antioxidants in Fodder Additives and Sheep Blood Serum by Amperometric and Biochemical Methods. Animals.

[39] Gusev I.V., Bogolyubova N.V., Rykov R.A., Levin G.N. (2019). Control of the Biochemical Status of Pigs and Cows.

[40] Romanov V.N., Bogolyubova N.V., Mishurov A.V. (2020). Multifunctional feed additive for increasing the adaptive capabilities and productivity growth of ruminants. Anim. Sci..

[41] Bogolyubova N.V., Rykov R.A., Romanov V.N. (2018). Assessment of the metabolic status of the body of bulls when using the energy-vitamin-mineral complex in nutrition. Anim. Husb..

[42] Niki E., Yoshida Y., Saito Y., Noguchi N. (2005). Lipid peroxidation: Mechanisms, inhibition, and biological effects. Biochem. Biophys. Res. Commun..

[43] Dyakova S.P. The relationship of lipid peroxidation with some hematological and biochemical parameters of blood is bright in different breeds. Proceedings of the International Scientific-Practical Conference “Free Radicals, Antioxidants and Animal Health”.

[44] Giro N.M., Egorova Z.G., Vornikova D.V., Zakharova N.B. (2013). Biochemical parameters of the blood serum of pigs and pork quality. Meat Ind..

[45] Nikolaev D.V., Kukushkina I.Y., Randelin D.A. (2011). Morphological and biochemical properties of the blood of pigs of Canadian selection. Bull. Altai State Agrar. Univ..

[46] Gu T., Shi J., Luo L., Li Z., Yang J., Cai G., Zheng E., Hong L., Wu Z. (2019). Study on hematological and biochemical characters of cloned Duroc pigs and their progeny. Animals.

[47] Kotenkova E.A., Kupaeva N.V. (2020). Assessment of the antioxidant potential of some by-products of pig slaughter. Food Ind..

