# Supplementary material for: Correlations between Antioxidant and Biochemical Parameters of Blood Serum of Duroc Breed Pigs
Source: Animals (Basel). 2021 Aug 13;11(8):2400. doi: 10.3390/ani11082400 (PMC8388761; doi:10.3390/ani11082400)
Supplement: Supplementary file 1 [file animals-11-02400-s001.zip › animals-1336188-supplementary.pdf]

animals

MDPI

Supplementary Materials to the manuscript
"Correlations Between Antioxidant and Biochemical Parameters of Blood Serum of Duroc Breed Pigs"

S.Yu. Zaitsev*, A.A. Belous, O.A. Voronina, A.A. Savina, R.A. Rykov, N.V. Bogolyubova :

Table S1. Correlations of biochemical parameters* of the blood serum of the Duroc breed piglets in group 1 (65 days of fattening).

	TP	A	G	A/G	Urea	Crt	Glucose	TG	Chol	ALT	AST	AST/ALT	ALP	Ca	P	Ca/P	Mg
TP	1.000	0.486	0.811	-0.499	0.099	0.229	-0.136	-0.055	0.460	0.466	0.201	-0.080	-0.052	-0.068	0.467	-0.344	0.422
A	0.486	1.000	-0.117	0.468	0.117	0.123	0.031	0.102	0.261	0.512	0.293	0.004	0.129	-0.075	0.398	-0.310	0.219
G	0.811	-0.117	1.000	-0.880	0.034	0.178	-0.176	-0.131	0.348	0.187	0.032	-0.094	-0.146	-0.027	0.265	-0.184	0.333
A/G	-0.499	0.468	-0.880	1.000	-0.063	-0.134	0.105	0.074	-0.214	0.068	0.060	0.029	0.143	-0.018	-0.070	0.036	-0.239
Urea	0.099	0.117	0.034	-0.063	1.000	0.231	0.187	0.146	0.053	-0.189	0.041	0.169	-0.041	0.335	0.031	0.145	0.343
Crt	0.229	0.123	0.178	-0.135	0.231	1.000	0.206	0.176	0.138	-0.072	0.098	0.088	0.323	0.149	-0.157	0.182	0.261
Glucose	-0.136	0.031	-0.176	0.105	0.187	0.206	1.000	0.015	-0.177	-0.370	0.058	0.260	0.322	0.324	-0.408	0.461	0.150
TG	-0.055	0.102	-0.131	0.074	0.146	0.176	0.015	1.000	0.101	0.019	-0.013	-0.017	0.285	-0.079	0.041	-0.086	0.006
Chol	0.460	0.261	0.348	-0.214	0.053	0.138	-0.177	0.101	1.000	0.471	0.089	-0.174	0.084	-0.298	0.531	-0.528	0.025
ALT	0.466	0.512	0.187	0.068	-0.189	-0.072	-0.370	0.019	0.471	1.000	0.190	-0.374	0.009	-0.317	0.512	-0.509	-0.097
AST	0.201	0.293	0.032	0.060	0.040	0.098	0.058	-0.013	0.089	0.190	1.000	0.806	-0.117	0.079	-0.050	0.084	0.198
AST/ALT	-0.080	0.004	-0.094	0.029	0.169	0.088	0.260	-0.017	-0.173	-0.374	0.806	1.000	-0.093	0.261	-0.334	0.368	0.285
ALP	-0.052	0.129	-0.146	0.143	-0.041	0.323	0.322	0.285	0.084	0.009	-0.117	-0.093	1.000	0.186	-0.025	0.110	0.033
Ca	-0.068	-0.075	-0.027	-0.018	0.335	0.149	0.324	-0.079	-0.298	-0.317	0.079	0.261	0.186	1.000	-0.365	0.770	0.372
P	0.467	0.398	0.265	-0.070	0.031	-0.157	-0.408	0.041	0.531	0.512	-0.050	-0.334	-0.025	-0.365	1.000	-0.862	0.017
Ca/P	-0.344	-0.310	-0.184	0.036	0.145	0.182	0.461	-0.086	-0.527	-0.509	0.084	0.368	0.110	0.770	-0.862	1.000	0.210
Mg	0.422	0.219	0.333	-0.239	0.343	0.261	0.150	0.006	0.025	-0.097	0.198	0.285	0.033	0.372	0.017	0.210	1.000

* TP - total protein, A - albumin, G - globulins, Crt - creatinine, TG - triglycerides, Chol - cholesterol, ALT - alanine aminotransferase, AST - aspartate aminotransferase, ALP - alkaline phosphatase, Ca – calcium, P – phosphorus, Mg – magnesium.

Table S2. Correlations of biochemical parameters of the blood serum of the Duroc breed piglets in group 2 (72 days of fattening).

	TP	A	G	A/G	Urea	Crt	Glucose	TG	Chol	ALT	AST	AST/ALT	ALP	Ca	P	Ca/P	Mg
TP	1.000	0.566	0.833	-0.544	0.457	0.481	-0.037	0.154	0.479	0.420	0.223	-0.006	0.016	-0.002	0.602	-0.526	0.566
A	0.566	1.000	0.015	0.341	0.322	0.230	0.102	0.117	0.296	0.594	0.224	-0.099	0.067	0.092	0.508	-0.360	0.834
G	0.833	0.015	1.000	-0.889	0.339	0.429	-0.113	0.109	0.383	0.111	0.120	0.060	-0.026	-0.064	0.389	-0.396	-0.544
A/G	-0.544	0.341	-0.889	1.000	-0.175	-0.297	0.099	-0.104	-0.267	0.098	-0.054	-0.120	0.0270	0.123	-0.178	0.270	0.457
Urea	0.457	0.322	0.339	-0.175	1.000	0.493	-0.078	0.050	0.246	0.117	0.307	0.239	-0.032	0.362	0.346	-0.076	0.481
Crt	0.481	0.230	0.429	-0.297	0.493	1.000	-0.089	0.074	0.468	0.199	0.219	0.109	0.084	-0.089	0.206	-0.266	-0.037
Glucose	-0.037	0.102	-0.113	0.099	-0.078	-0.089	1.000	-0.023	0.109	-0.081	-0.090	-0.105	0.339	0.001	0.091	-0.062	0.154
TG	0.154	0.117	0.109	-0.104	0.050	0.074	-0.023	1.000	0.170	0.217	0.146	0.019	0.262	-0.031	0.065	-0.103	0.479
Chol	0.479	0.296	0.387	-0.266	0.246	0.468	0.109	0.170	1.000	0.370	0.189	-0.011	0.223	-0.057	0.352	-0.352	0.420
ALT	0.420	0.595	0.111	0.098	0.117	0.199	-0.081	0.217	0.370	1.000	0.269	-0.222	0.084	-0.037	0.323	-0.288	0.223
AST	0.223	0.224	0.120	-0.054	0.307	0.219	-0.090	0.146	0.190	0.268	1.000	0.866	-0.338	-0.069	0.143	-0.161	-0.006
AST/ALT	-0.006	-0.099	0.060	-0.120	0.239	0.109	-0.105	0.019	-0.011	-0.222	0.866	1.000	-0.393	-0.079	-0.019	-0.032	0.016
ALP	0.016	0.067	-0.026	0.027	-0.032	0.084	0.339	0.262	0.223	0.0837	-0.338	-0.393	1.000	0.125	0.122	-0.018	-0.002
Ca	-0.002	0.092	-0.064	0.123	0.362	-0.089	0.001	-0.031	-0.057	-0.037	-0.070	-0.080	0.125	1.000	0.065	0.595	0.602
P	0.602	0.508	0.389	-0.178	0.346	0.206	0.091	0.065	0.352	0.323	0.143	-0.019	0.122	0.065	1.000	-0.748	-0.526
Ca/P	-0.526	-0.360	-0.396	0.270	-0.076	-0.266	-0.062	-0.103	-0.352	-0.288	-0.161	-0.032	-0.018	0.595	-0.748	1.000	0.775
Mg	0.775	0.512	0.596	-0.360	0.526	0.374	-0.099	-0.033	0.371	0.313	0.329	0.191	-0.042	0.113	0.563	-0.386	1.000

* TP - total protein, A - albumin, G - globulins, Crt - creatinine, TG - triglycerides, Chol - cholesterol, ALT - alanine aminotransferase, AST - aspartate aminotransferase, ALP - alkaline phosphatase, Ca – calcium, P – phosphorus, Mg – magnesium.

Table S3. Correlations of biochemical parameters of the blood serum of the Duroc breed piglets in group 3 (100 days of fattening).

	TP	A	G	A/G	Urea	Crt	Glucose	TG	Chol	ALT	AST	AST/ALT	ALP	Ca	P	Ca/P	Mg
**TP**	1.000	0.159	0.868	-0.623	-0.187	-0.243	0.499	0.542	-0.136	0.762	0.721	-0.544	0.651	0.356	0.628	0.017	0.999
**A**	0.159	1.000	-0.351	0.665	0.320	0.019	0.440	0.311	0.429	0.549	0.187	-0.152	0.642	0.238	0.720	-0.237	0.159
**G**	0.868	-0.351	1.000	-0.925	-0.339	-0.240	0.252	0.358	-0.345	0.446	0.590	-0.440	0.295	0.218	0.234	0.135	0.868
**A/G**	-0.623	0.665	-0.925	1.000	0.360	0.120	-0.052	-0.206	0.408	-0.149	-0.380	0.270	0.031	-0.084	0.119	-0.261	-0.623
**Urea**	-0.187	0.320	-0.339	0.360	1.000	-0.185	-0.289	0.667	0.496	-0.072	-0.287	0.488	-0.282	-0.682	0.103	0.500	-0.187
**Crt**	-0.243	0.019	-0.240	0.120	-0.185	1.000	0.272	-0.261	0.221	-0.157	-0.304	0.341	-0.122	0.144	-0.298	-0.140	-0.243
**Glucose**	0.499	0.440	0.252	-0.052	-0.289	0.272	1.000	0.251	-0.422	0.840	0.794	-0.548	0.478	0.570	0.222	-0.019	0.499
**TG**	0.542	0.311	0.358	-0.206	0.667	-0.261	0.251	1.000	0.147	0.575	0.403	-0.119	0.173	-0.203	0.375	0.646	0.542
**Chol**	-0.136	0.429	-0.345	0.408	0.496	0.221	-0.422	0.147	1.000	-0.256	-0.653	0.485	0.204	-0.228	0.463	-0.154	-0.136
**ALT**	0.762	0.549	0.446	-0.149	-0.072	-0.157	0.840	0.575	-0.256	1.000	0.889	-0.725	0.712	0.554	0.575	0.141	0.762
**AST**	0.721	0.187	0.590	-0.380	-0.287	-0.304	0.794	0.403	-0.653	0.889	1.000	-0.793	0.476	0.506	0.279	0.170	0.721
**AST/ALT**	-0.544	-0.152	-0.440	0.270	0.488	0.341	-0.548	-0.119	0.485	-0.725	-0.793	1.000	-0.656	-0.834	-0.325	-0.123	-0.544
**ALP**	0.651	0.642	0.295	0.031	-0.282	-0.122	0.478	0.173	0.204	0.712	0.476	-0.656	1.000	0.727	0.873	-0.333	0.651
**Ca**	0.356	0.238	0.218	-0.084	-0.682	0.144	0.570	-0.203	-0.228	0.554	0.506	-0.834	0.727	1.000	0.302	-0.169	0.356
**P**	0.628	0.720	0.234	0.119	0.103	-0.298	0.222	0.375	0.463	0.575	0.279	-0.325	0.873	0.302	1.000	-0.332	0.628
**Ca/P**	0.017	-0.237	0.135	-0.261	0.500	-0.140	-0.019	0.646	-0.154	0.141	0.170	-0.123	-0.333	-0.169	-0.332	1.000	0.017
**Mg**	0.999	0.159	0.868	-0.623	-0.187	-0.243	0.499	0.542	-0.136	0.762	0.721	-0.544	0.651	0.356	0.628	0.017	1.000

* TP - total protein, A - albumin, G - globulins, Crt - creatinine, TG - triglycerides, Chol - cholesterol, ALT - alanine aminotransferase, AST - aspartate aminotransferase, ALP - alkaline phosphatase, Ca – calcium, P – phosphorus, Mg – magnesium.
